# Assessment of Macular Function during Vitrectomy: New Approach Using Intraoperative Focal Macular Electroretinograms

**DOI:** 10.1371/journal.pone.0144627

**Published:** 2015-12-10

**Authors:** Celso Soiti Matsumoto, Kei Shinoda, Gaku Terauchi, Harue Matsumoto, Atsushi Mizota, Yozo Miyake

**Affiliations:** 1 Department of Ophthalmology, Teikyo University School of Medicine, Kaga 2-11-1, Itabashi-ku, Tokyo 173–8605, Japan; 2 Matsumoto Eye Clinic, Tokushima, Japan; 3 Aichi Medical University, 1–1 Yazakokarimata, Nagakute, Aichi, 480–1195, Japan; University of Florida, UNITED STATES

## Abstract

**Purpose:**

To describe a new technique to record focal macular electroretinograms (FMERGs) during vitrectomy to assess macular function.

**Methods:**

Intraoperative FMERGs (iFMERGs) were recorded in ten patients (10 eyes) who undergo vitrectomy. iFMERGs were elicited by focal macular stimulation. The stimulus light was directed to the macular area through a 25 gauge (25G) glass fiber optic bundle. Background light was delivered through a dual chandelier-type light fiber probe. Focal macular responses elicited with combinations of stimulus and background luminances were analyzed.

**Results:**

A stimulus luminance that was approximately 1.75 log units brighter than the background light was able to elicit focal macular responses that were not contaminated by stray light responses. Thus, a stimulus luminance of 160 cd/m^2^ delivered on a background of 3 cd/m^2^ elicited iFMEGs from only the stimulated area. This combination of stimulus and background luminances did not elicit a response when the stimulus was projected onto the optic nerve head. The iFMERGs elicited by a 10° stimulus with a duration of 100 ms and an interstimulus interval of 150 ms consisted of an a-, b-, and d-waves, the oscillatory potentials, and the photopic negative response (PhNR).

**Conclusions:**

Focal ERGs with all components can be recorded from the macula and other retinal areas during vitreous surgery. This new technique will allow surgeons to assess the function of focal areas of the retina intraoperatively.

## Introduction

In 1981, Miyake et al developed a system to record focal macular electroretinograms (FMERGs) from humans [1]. In their system, a 5°, 10°, or 15° stimulus was delivered to a focal retinal area while its position was continuously monitored with a fundus camera. The recorded FMERGs consisted of a-, b-, and d-waves, the oscillatory potentials (OPs), and the photopic negative response (PhNR). Since then, FMERGs have been used to assess the physiological condition of retinal neural cells including the photoreceptors in the macular area [2–7].

There are essentially three ways to deliver a stimulus to a specific area of the retina under fundus observation; through a fundus camera as is done with the Miyake system [2–7], through a slit-lamp microscope[8,9], or through a direct ophthalmoscope [10]. All of these methods are non-invasive and easily performed in the clinic, and several investigators have evaluated the macular function in different macular diseases with one of these methods. The assessments were made before and after therapeutic interventions including vitreous surgery [11–18], and were also made to determine the functional properties of the retina of eyes with hereditary macular dystrophies [19–21].

Although vitreous surgery has improved considerably [22], the surgery is invasive and little is known on whether retinal functional is affected by the vitrectomy procedures. Post-vitrectomy assessments of retinal function have shown that the function can occasionally be altered. For example, Miyake et al used full-field ERGs to examine the retina after the vitrectomy, and they reported time-dependent alterations in retinal function during the vitrectomy [23,24]. However, techniques are not available to examine macular function during the vitrectomy. This prompted us to try to develop a technique to record FMERGs during vitrectomy.

Thus, the purpose of this study was to develop a new method to record FMERGs during surgery. We have called the responses recorded the intraocular FMERGs (iFMERGs). If successful, this technique should allow clinicians to evaluate the retinal function intraoperatively, and thus evaluate the effects of manipulating the macular area, the effects of adjunctive intravitreal drugs, and the effects of potentially harmful procedures online.

## Subjects and Methods

### Subjects

Ten patients who were scheduled to undergo vitreous surgery were studied. There were 6 men and 4 women whose mean age was 61 ± 5 years (± standard deviation) with a range of 49 to 75 years. Seven patients with macular sparing rhegmatogenous retinal detachment (RRD) and three patients with intraocular lens dislocation were included. Two among RRD eyes had mild rhegmatogenous vitreous hemorrhage. All patients had no macular diseases throughout the study.

### Methods

The procedures used conformed to the tenets of the Declaration of Helsinki. The study was a observational case series with approval of the Ethics Committee of the Teikyo University School of Medicine (Study ID Number: 10-033-2) and written informed consent was obtained from all participants to participate in research.

The eyes were anesthetized by a subtenon injection of 3 to 4 ml of 2% lidocaine, and the pupils were dilated with topical tropicamide (0.5%) and phenylephrine hydrochloride (5%). The non-stimulated eye was covered with the surgical drape.

Standardized 3 port pars plana vitrectomy was performed with a 25 gauge vitrectomy system [22]. The temperature of the operating room was kept at 25°C. The vitreous cutter was controlled by a vitreous surgical system (Constellation Vision System^Ⓡ^, Alcon Surgical, Fort Worth, Texas, USA). The intraocular pressure (IOP) was kept at 30 mmHg during the surgery with the IOP control system that was integrated into the surgical system. Endoillumination was obtained from a chandelier-type fiber optic probe (29G, Oshima Dual Chandelier, Synergetics^™^ Inc, MO, USA). All iFMERG recordings were performed during surgery after a 5 minute adaptation to the operating room lights.

### Photopic stimuli

An operating microscope (Model M844^®^, Leica Microsystems, Weltzer, Germany) combined with a wide angle fundus observation system (BIOM^®^, Oculus, Weltzer, Germany) was used to observe the fundus during the surgery and the recordings of the iFMERGs. High flux, white light emitting diodes (LEDs, OSW4XME3C1E, Optosupply, Taiwan) were used for the light stimuli, and the stimuli were delivered to the macular area by a 25G directional glass optic fiber cable (25G Directional Laser Probe, Synergetics^™^, MO, USA). The size of the stimulus spot was approximately 1015°, and the size was controlled by the distance between the tip of the endoprobe and the retinal surface. Anatomically, optic nerve head in normal eyes has approximately 5 degree in the largest diameter. The stimulus spot size was determined as 2 3 times of optic nerve head. The image from a CCD camera mounted in the operating microscope was displayed in the LCD monitor and the stimulus spot size was adjusted according to the indication by one of the operation staffs who was comparing the spot size and disc size on the display during surgery. To keep size constant the surgeon fix the hands and fingers in patient head and eye surface to maintain the distance between the probe and retinal surface. The stimulation system was designed so that the incidence of the stimulus light on the retina was parallel to the visual axis to avoid the Stiles-Crawford effect (SCE) [25].

**Fig 1:**
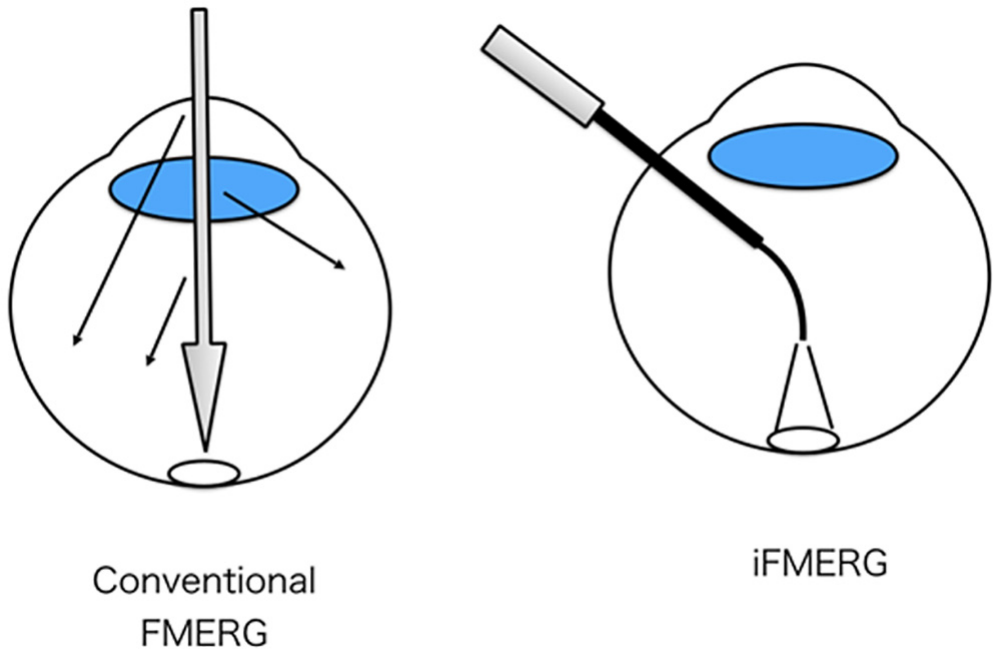
Schematic diagrams of the two stimulus conditions. The stimuli used for conventional focal macular electroretinograms (FMERGs; left) and the intraoperative iFMERG (right) are shown. Because the focal stimuli using an endolaser probe was directly delivered to the macula without passing through the ocular media, the stray light effect is minimized.

The iFMERGs were elicited by 4-Hz rectangular stimuli (100-ms light on and 150-ms light off), and the luminance of the constant background illumination was 3 cd/m^2^. The background illumination was used to depress the sensitivity of the area surrounding the focal stimulus and thus reduced the stray light responses. The stray light effect was also minimized because the focal stimuli were delivered to the macula with an endolaser probe without passing through the ocular media which would lead to scattered light (Fig 1). In addition, background light from high flux LED chandelier light source illuminated a wide area of the retinal surface which also reduced the stray light effect.

### Recording intraoperative focal macular electroretinograms (iFMERGs)

**Fig 2:**
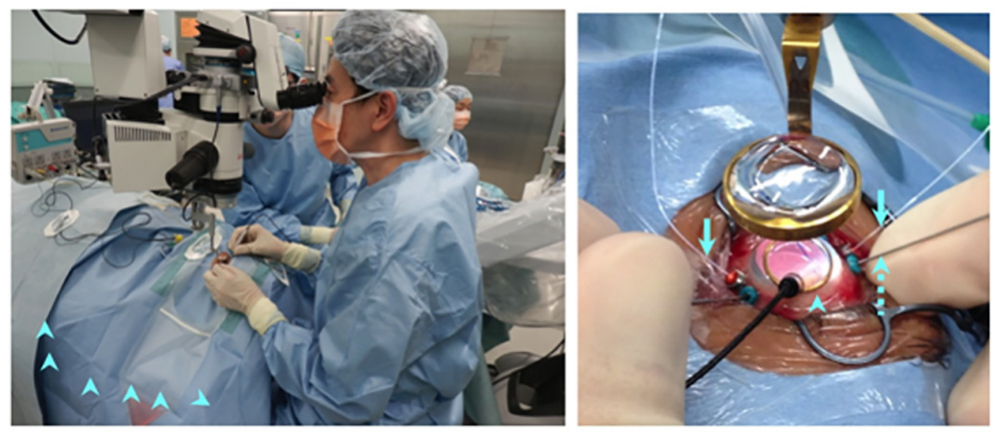
Photograph showing operating condition during the recording of the iFMERGs. A gold-foil monopolar contact lens electrode (Mayo Corporation, Nagoya, Japan) was sterilized and placed on the cornea (arrowhead) of the examined eye to pick up the iFMERGs. A dual port 29G Oshima chandelier light probe (Synergetics, O’Fallon, MO, USA) was used for background light (arrow). A 25G flexible directional fiber probe (Synergetics, O’Fallon, MO, USA) was used to deliver the stimuli to the retina (dotted line arrow).

A gold foil monopolar contact lens (Mayo Corporation, Nagoya, Japan) was sterilized and placed on the cornea of the examined eye to pick up the iFMERGs (Fig 2). The CL (with 0 diopter) was placed on the cornea, and a high power non-contact lens was located over the cornea that provided the wide viewing of the fundus.

The reference silver plate electrode was placed on the forehead at the midline, and the ground electrode was attached to the ipsilateral ear lobe.

Because the light emitted from the endo-illumination probe is too bright and saturate the retina photopically in an ununiformed way during vitreous surgery, we stop the surgery and adapted the eye under room lights for a period of 5 minutes for each session of ERG recording. The iFMERG was recorded just after core vitrectomy.

After centering and adjusting the light spot size on the retina, the stimuli were turned on. The iFMERGs were amplified and averaged with a bioamplifier (MEB-9404, Nihon Kohden Corporation, Tokyo, Japan). One hundred responses were averaged, and the sampling rate was 10 kHz, i.e., every 0.1 ms. The responses were filtered between 20 to 200 Hz by a hardwired band pass filter for recording the a-, b-, and d- waves of the photopic FMERGs. The OPs and PhNR were recorded with the band pass filter set at 100 to 500 Hz. A narrow filter for 50 Hz was used to improve signal to noise level. The noise level with the electrode placed on the cornea with no stimulus was less than 0.1 μV.

The amplitude of the a-wave was measured from the baseline to the trough of the a-wave, and the amplitude of the b-wave was measured from the trough of the a-wave to the following positive peak. The amplitude of the d-wave was measured from the trough just preceding the d-wave to the positive peak after the stimulus offset.

## Results

The iFMERG recordings for one session took approximately 25 seconds because single record was obtained by a light stimulus of 100-ms with a 150-ms light off interval and one hundred signals were averaged to record an iFMERG waveform. No complications related to the iFMERG recordings were observed. A flickering light was perceived by some patients during the recordings but this did not distract from the surgery. No additional instruments were required for the iFMERG recordings.

The analysis of the iFMERG waveforms recorded just after core vitrectomy using the stimulus of 10 degree spot size with 160 cd/m^2, frequency of 4Hz, and duration of 100ms showed the average amplitude(standard deviation) for each component were 2.50(0.45) μV for the a-wave, 6.0(1.18) μV for the b-wave, and 2.57(0.39) μV for the d-wave.

### Waveform of intraoperative focal macular electroretinogram (iFMERG)

**Fig 3:**
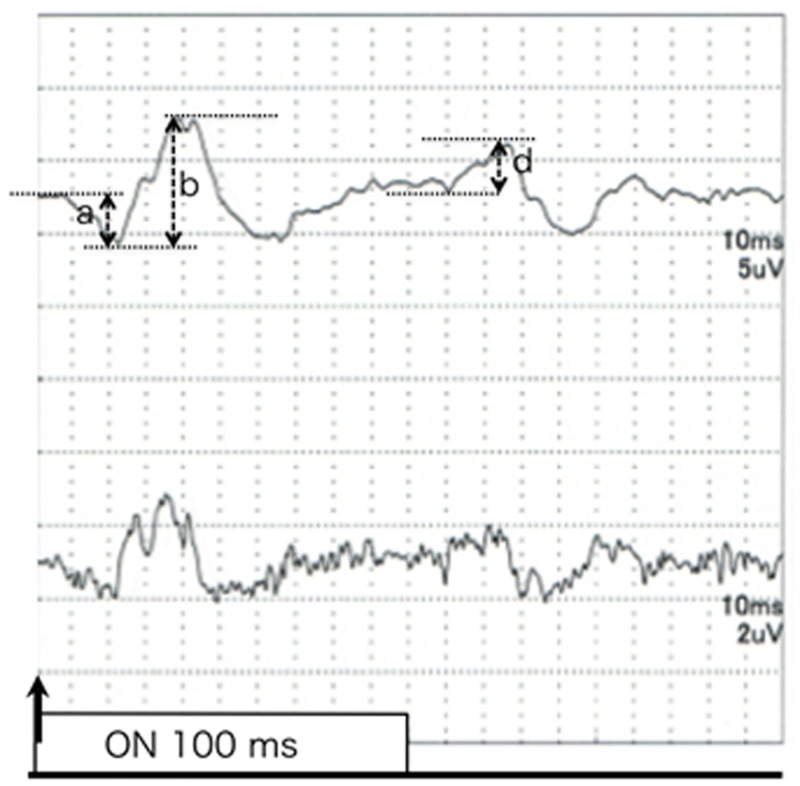
iFMERGs recorded from a normal eye. Upper: Waveform of iFMERG elicited by a long duration stimulus. Bottom: Oscillatory potentials of focal macular ERGs elicited by a 10° diameter stimulus.

**Fig 4:**
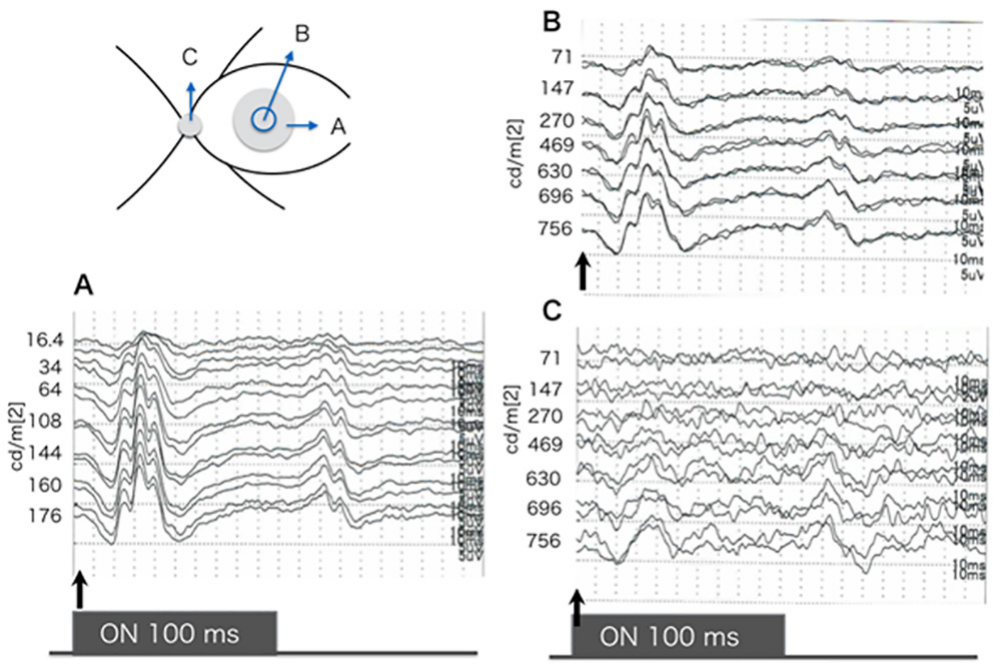
Focal macular ERGs elicited by different stimulus intensities from a normal subject. A: A 10° spot of was projected onto the macula. B: A 5° stimulus spot was projected onto the macula. C: The round stimulus was projected onto the optic disc. The spot size was controlled to correspond to the optic disc. The stimulus duration is 100 ms and the luminance of the constant background illumination is 3.0 cd/m^2^. An increase in the amplitudes of each component is observed with increasing luminance but the implicit times appear to be constant. The iFMERGs recorded by the stimulus projected on the optic nerve head were non-recordable when the intensity was ≤270 cd/m^2^ indicating that this stimulus intensity could elicit a focal response only from the macula with negligible stray light effect.

The iFMERGs recorded from a normal subject had the different ERG components and are shown in Figs 3 and 4. The waveform and components of the iFMERGs were similar to those of the conventional FMERGs and full-field photopic ERGs. The a- and b-waves, OPs, photopic negative response (PhNR), and d-wave elicited by the long duration stimuli can be seen.

### Stimulus intensity-response series of intraoperative focal macular electroretinograms

Intraoperative FMERGs were elicited by different stimulus intensities from an eye with macula-spared retinal detachment (Fig 4A), rhegmatogeneous vitreous hemorrhage (Fig 4B), and IOL luxation (Fig 4C). An increase in the amplitude of each component of the iFMERGs was observed with increasing luminance but the implicit times appeared to be relatively constant (Fig 4).

**Fig 5:**
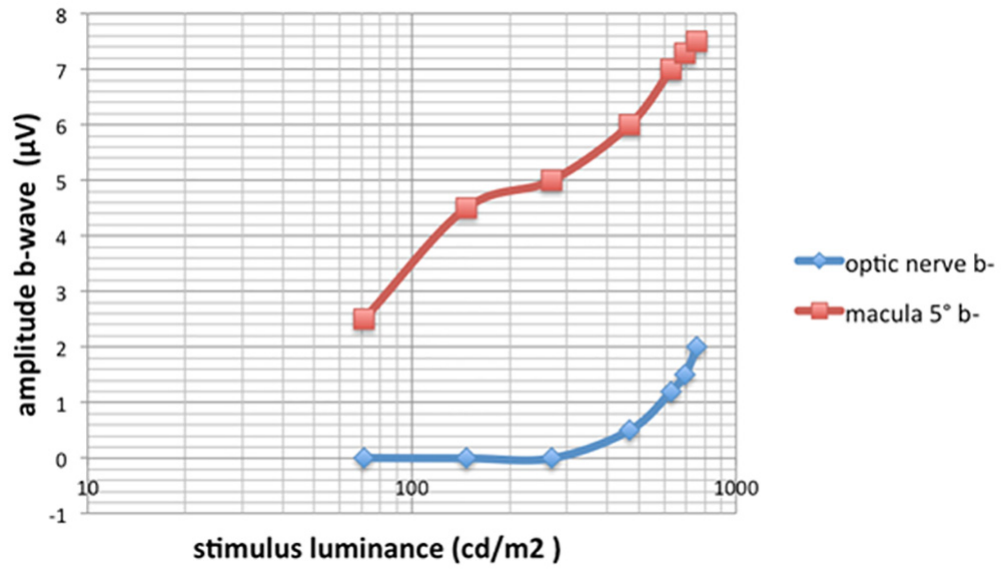
Stimulus intensity vs b-wave amplitude for stimulus spot projected onto the macula and onto the optic nerve head. The size of the stimulus was 5°. A stimulus spot that was approximately 1.75 log unit brighter than the background light was able to elicit a focal macular response.

The position of the light spot on the macular area was monitored during all of the recordings. The stimulus spot is 10°in diameter. The stimulus duration is 100 ms and the luminance of the constant background illumination is 3.0 cd/m^2^. An increase in the amplitudes of each component is observed with increasing luminance but the implicit times appear to be constant. When the stimulus was projected onto the optic nerve head, an iFMERG was not recorded for intensities ≤270 cd/m^2^ indicating that this stimulus intensity can be used to elicit a focal response only from the macula with negligible effects of stray light (Figs 4C and 5). An approximately 1.75 log unit brighter stimulus light than the background light was able to elicit focal macular responses.

The intensity of 270 cd/m^2^ was almost one order brighter compared to 30 cd/m^2^ which has been used for the conventional fundus camera type FMERG system^1^. This is probably because the stimulus light in our system skips over the ocular media such as cornea, lens, and anterior vitreous, therefore free from their stray effect.

### Intraoperative focal macular electroretinograms (iFMERGs) elicited by different stimulus durations

**Fig 6:**
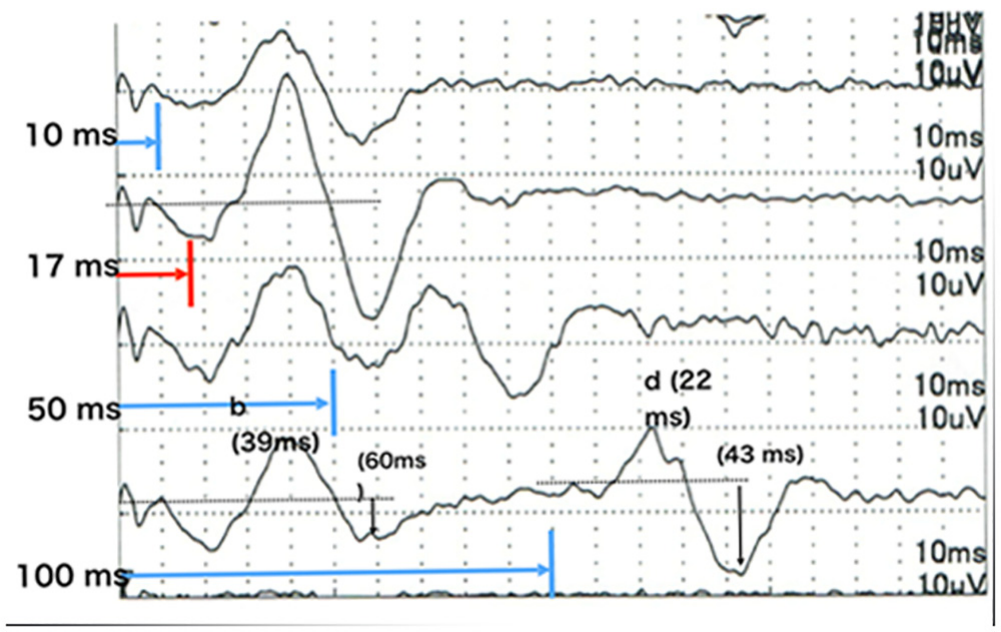
Effect of stimulus duration on intraoperative focal macular electroretinograms from a normal eye. With shorter stimulus durations, the total light energy was relative low therefore the amplitude of b-wave was relative small. But with shorter duration the b- and d-waves (on and off responses) became closer and merged with stimuli intervals less than 17 ms.

iFMERGs were recorded with constant stimulus frequency of 4 Hz but with stimulus durations of 10, 17, 50, 100 ms (Fig 6). With shorter stimulus durations, the total light energy was relative low therefore the amplitude of b-wave was relative small. But with shorter durations, the off responses, the d-waves, move closer to the b-wave and merged with a duration of <17 ms. Therefore, the b-wave amplitude and the PhNR were largest when the stimulus duration was 17 ms.

### Intraoperative focal macular electroretinogram (iFMERG) from different retinal areas

**Fig 7:**
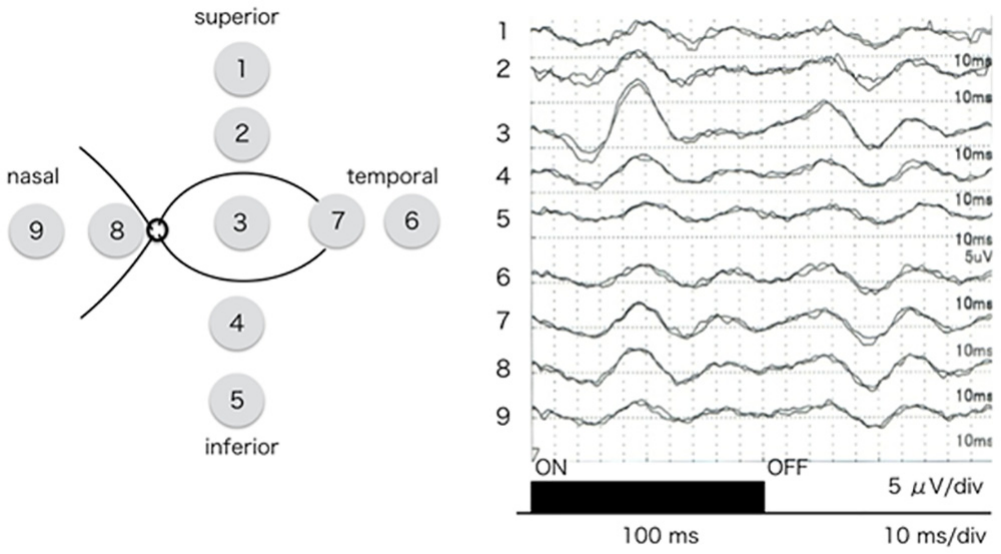
Intraoperative focal macular electroretinograms recorded from different retinal sites. iFMERGs were elicited by circular stimuli placed at 9 sites on the retina. The a-, b-, and d-waves in the central area are larger than those in the peripheral area. The implicit time of each component was shorter in the central area and longer at the more peripheral areas.

iFMERGs were elicited from stimulating different retinal locations (Fig 7).The a-, b-, and d-waves were larger in the central macular area and decreased gradually at more peripheral areas. The implicit time of each component was shorter in the central area and was longer at more peripheral areas.

## Discussion

Vitreous surgery has become a relatively common procedure for many diseases, and the success rate has increased considerable. Because the vitreoretinal condition is markedly changed during the intraocular surgical procedures, it is reasonable to question whether there may be pathological effects on the vitreoretinal physiology. However, little is unknown about the functional changes of the retina during the intraocular surgery.

We have investigated the visual sensations experienced by patients during vitrectomy, and found that many patients had visual phosphenes [26–28]. Moreover, patients with more complex visual sensations, e.g., perception of intraocular surgical instruments, had better visual improvements after the surgery. But these observations were obtained by subjective testing, and no quantitative measurements could be made. Miyake et al recorded 30-Hz flicker ERG using a contact lens electrode with a built-in light-emitting diode during eye surgery [23,24,29]. However, these studies examined the response to full-field stimulation, and so far no technique is available for recording from focal areas of the retina during surgery. The ability to record iFMERG will make it possible to evaluate different areas of the retina objectively during the course of vitrectomy.

All of the instruments used to record the iFMERGs are used during standard vitreous surgery; the operating microscope and wide viewing system to view the fundus, chandelier lightning system for the background light, and the endolaser probe for the stimulus spot. The only thing we needed to change was the contact lens; replacing the one used for the surgery with one with gold foil electrodes to pick up the iFMERGs.

The stimulus intensity in the conventional fundus camera type FMERG system is 30 cd/m^2^. The brighter light is unavoidable of stray light because the stimulus light goes through ocular media. However, in the iFMERG system, the stimulus light directly falls on the macula without going through the ocular media. Therefore such brighter light can be used for local stimulus.

The present results should be carefully interpreted because the recording system of the iFMERGs is different from that of conventional FMERGs. First, the FMERGs that were elicited by the stimulus projected onto the optic nerve head became non-recordable only when the intensity was ≤270 cd/m^2^. Previous study using multifocal ERG have been showed that optic nerve head has a high reflectance properties compared to others areas of the retina [30]. Taken together, our results indicates that higher stimulus intensities projected on the macula will be contaminated by stray light responses.

Second, the stimulus light emitted from the endolaser probe was not parallel. Thus, some degree of the SCE must be considered. We believe that by placing the laser probe close to the retina will minimized the SCE but the degree of the SCE was not determined.

Third, the influence of adaptation on the FMERG has not been determined definitively. Eyes undergoing vitrectomy are normally exposed to light by endoillumination, illumination by the operating microscope, and chandelier lighting. The durations of these exposures varied among the patients, and the 5 min of light-adaptation before the beginning of the iFMERG recordings may not have been sufficient to bring all of the retinas to the same adaptation level.

The iFMERG system has several drawbacks. It takes several seconds for the repetitive recordings which would increase the operation time. In addition, the procedures require moving the instruments in and out through the trocars. To minimize IOP fluctuation and vitreous incarceration into trocars, we recommend using valved trocars. In spite of all of these negative conditions, we did not have any evidence of retinal damage or intra- and postoperative complications. However, it should be kept in mind that excessive light exposure during vitrectomy can be stressful on the diseased macula although the light used for the iFMERG is much darker compared to the endoillumination used for retinal visualization during standard vitrectomy therefore considered to be no harmful to the retina.

In conclusion, we present an intraoperative FMERG recording system that can be used during vitreous surgery. This system can record ERGs from focal areas of the retina with minimal contamination by stray light responses. This system will enable real time assessment on the local retinal function and a layer-by-layer analysis of the retina during surgery.
